# Treatment of Lethal Caffeine Overdose with Haemodialysis: A Case Report and Review

**DOI:** 10.2478/jccm-2022-0019

**Published:** 2022-11-12

**Authors:** Christian C Toquica Gahona, Ashwin Kodagnur Bharadwaj, Monarch Shah, Umesh Bhagat, Paul Sterman, William Vasquez

**Affiliations:** 1 Saint Peter's University Hospital, New Brunswick, NJ, USA

**Keywords:** caffeine, drug overdose, critical care, haemodialysis

## Abstract

Caffeine, chemically 1,3,7-trimethylxanthine, is the most widely consumed central nervous system stimulant in the world with pleiotropic effects on the cardiovascular, pulmonary, and renal systems. The advent of over the counter (OTC) caffeine formulations has opened the window for potential toxicity, either by inadvertent or intentional overdosing. We present the case of a patient who attempted suicide by caffeine overdose treated with emergent haemodialysis and a review of the literature.

## Introduction

Caffeine, chemically 1,3,7-trimethylxanthine, is a plant-based alkaloid found in about 60 plant species [1]. It is the most widely consumed central nervous system stimulant in the world with patronage by almost 80% of humanity. The beverages, “coffee”, “tea” and soft drinks remain the primary conduits for caffeine into the human body [2], however, energy drinks containing caffeine are growing in popularity in the US [3, 4]. Aside from the nervous system, caffeine has effects on multiple other organ systems with the effects on the cardiovascular, pulmonary, gastrointestinal and renal systems being the most clinically relevant [5]. Here we present a case of attempted suicide by caffeine overdose.

### Epidemiology

The advent of over the counter [OTC] caffeine formulations has opened the window for potential toxicity, either by inadvertent or intentional overdosing. Caffeine consumption is widely prevalent in all races and ethnic groups, with no scientific data showing preference in any groups [3, 6].

In the United States for the year 2018 the American Association of Poison Control Centres [AAPCC] reported 1592 single exposures to caffeine-containing energy drinks. From those 6 had major outcomes defined as signs or symptoms that were life-threatening or resulted in significant residual disability or disfigurement. No deaths were reported. Most exposures from caffeinated energy drinks happened in children younger than 6 years of age. With adults 20 years and older representing the second highest number of exposures. Gender was not included in this report. Additionally, the AAPCC also reported 2849 cases of caffeine exposure from street drugs use with 21 major outcomes and 2 reported deaths [7].

## Methods

Original publications and case reports of caffeine-overdose were identified from searching PUBMED, Embase, Ovid and GOOGLE-SCHOLAR and review of references of related articles. This search strategy located 21 well-documented reports of caffeine overdose in adults treated with dialysis techniques. Details pertaining to the serum caffeine levels, clinical presentation and treatment modalities were reviewed and extracted. Similarly, a review of the pharmacokinetics of caffeine was performed and discussed in the article.

## Case Presentation

32-year-old male with no past medical history was brought to the emergency room by emergency medical services 1 hour after a suicidal attempt from ingestion of 200 pills of 250mg of caffeine (Total 50g). He developed nausea and vomited food particles without any blood or bile. Initially he was feeling restless, with no complains of fever, tremors, or changes in the vision, chest pain, palpitations, shortness of breath or diarrhoea. Initially he did not mention his pill ingestion until one hour after admission, about two hours after the initial intake.

On admission physical examination showed a Glasgow coma scale (GSC) of 15, blood pressure 126/72 mmHg heart rate: 135 beats/min, respiration rate: 29 beaths/min, oxygen saturation 96% on Room air. Temperature: 97.4 Fahrenheit.

Patient agitated in distress, His pupils were equal, round, and reactive to light, cardiovascular exam evidenced tachycardia, normal S1 and S2, no murmurs, rubs or gallops, no wheezing or rales on lung examination, no skin rashes or flushing, no muscle weakness, or sensory deficits appreciated.

**Fig.1:**
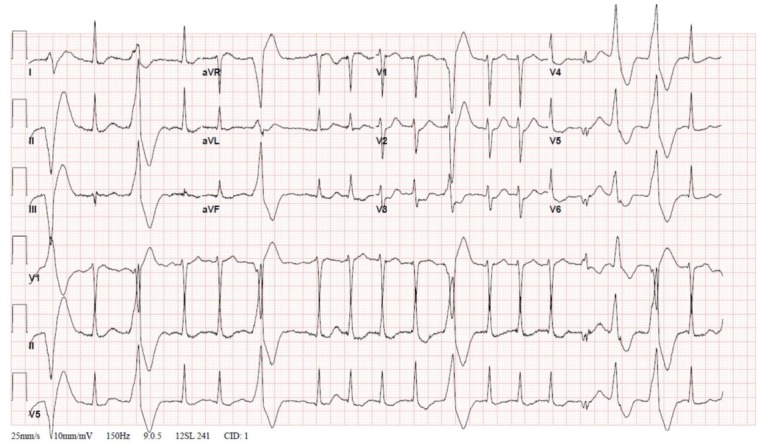
Electrocardiogram showing tachycardia with an undetermined rhithm, HR-108/min, normal axis, ST segment depression in V2-V3, and multiple premature ventricular contractions.

Initial EKG showed tachycardia with an undetermined rhythm, ST segment depression in V2-V3, and multiple premature ventricular contractions, (Figure 1). The patient was transferred to the ICU for monitoring, given the risk of development of life-threatening complication.

On initial investigations patient had leucocytosis 16.9×109/l (4.0–11.0), with 57.7% neutrophils, hyperglycaemia of 197mg/dL (74–106), hypokalaemia 2mmol/L (3.5-5.1 mmol/L), hypocalcaemia of 6.5mg/ dL (8.4-10.0 mg/dL), hypophosphatemia of 2.5 mg/dL (2.7–4.5), severe lactatemia with serum lactate level of 5.2 mmol/L (0.5-2.0 mmol/L), urine toxicology screen and two sets of troponins were negative (Table 1).

**Table 1:** Laboratory results during hospital course.

Laboratory test	Admission day	Day 2	Day 3	Day 4
WBC (4.0-11.0 10 3/cumm)	16.9	16	7.3	
ABS lymphocytes	5.6	2.6		
HGB (12.0-16.0 g/dL)	14.1	13.2	13.6	
Platelet count (150-400 10 3/cumm)	335	263	236	
BUN (9-28 mg/dL)	11	7	12	13
Creatinine (0.52-1.04 mg/dL)	0.72	0.92	0.98	1.06
AST (SGOT) (14-36 U/L)	20			
ALT (SGPT) (9-52 U/L)	23			
CO2 (21-33 mmol/L)	19	21	24	31
Anion gap	15	17	8	6
Lactic acid (0.5-2.0 mmol/L)	5.2-4.7-1.6	0.6		
BNP *10.0-100.0 pg/mL)	<10			
Troponin-I (< 0.12 ng/mL)	<0.03	<0.03		
Protein (6.0-8.0 g/dL)	5.7			
Albumin (3.2-4.6 g/dL)	3.7			
Calcium (8.4-10.0 mg/dL)	6.5	8.4	8.5	8.9
Glucose (82-115 mg/dL)	197	112	90	87
Total bilirubin (0.1-1.2 mg/dL)	0.4			
ALK phosphatase (53-141 U/L)	43			
Sodium (136-145 mmol/L)	140	138	139	138
Potassium (3.5-5.1 mmol/L)	2	3.6	4	4.3
Chloride (99-112 mmol/L)	106	104	104	101
Magnesium (1.60-2.66 mg/dL)	1.2	1.5	1.4	1.7
Caffeine in serum (8.0-20.0 mg/L)	>90	6.7		
Alcohol (ethanol)	<10 mg/dL = None Detected			
Acetaminophen	Negative			
Anphetamine, urine	Negative			
Barbituates, urine	Negative			
Benzodiazepine, urine	Negative			
Cocaine, urine	Negative			
Methadone, urine	Negative			
Opiates, urine	Negative			
PCP, urine	Negative			
Salicylate, urine	Negative			
THC	Negative			

* BNP: B-type Natriuretic peptide

**Fig. 2:**
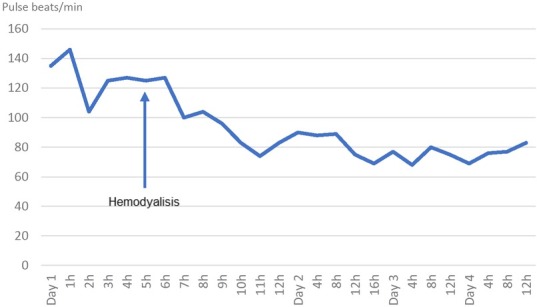
Relationship of heart rate in response of initiation of treatment with hemodialysis. Arrow showing time of initiation of hemodialysis.

Toxicology and nephrology were consulted, and decision was made to perform emergent haemodialysis (HD) which he underwent for 4 hours. After 1 hour of initiating treatment with HD the heart rate and restlessness subsided, and after 8 hours of hospital admission his vital signs returned to normal and no betablockers had to be used (Figure 2). A repeat EKG done after dialysis was performed with resolution of the previous findings.

No activated charcoal was administered given that the time of ingestion was unclear during the initial evaluation. A foley catheter was placed to ensure adequate fluid balance. Given the diuretic effect caused by caffeine the patient had large amounts of urine output, a total of 5.5L in the first 24 hours. To prevent hypovolemia, he received intravenous crystalloids to keep up with his losses in a 1:1 ratio. After the first 24 hours his urine output decreased, and the intravenous fluids were reduced accordingly.

Other medications used during hospitalization included: Metoclopramide given for nausea, pantoprazole for protection o f gastric mucosae, electrolyte replacement (potassium, calcium, magnesium, phosphate), and enoxaparin subcutaneously for venous thromboembolism prophylaxis.

The patient had a Psychiatric evaluation and was started on Bupropion 150mg once daily on day 3 of hospital course. Ultimately on day 4 the patient agreed for a voluntary hospitalization and was transferred to an inpatient psychiatric unit.

## Discussion

Caffeine is rapidly and almost completely (~ 99%) absorbed from the small intestine reaching plasma concentration 90 to 120 minutes after ingestion, with non-significant first pass metabolism in the liver [8]. The lipophilic nature of caffeine allows it to penetrate all tissues including the blood-brain barrier [9]. Caffeine is inactivated by CYP1A2 by linear first order kinetics which can become saturated, subsequently being metabolized by a zero-order kinetics at higher doses. Paraxanthine is the major metabolite of caffeine bio-transformation, while theobromine and theophylline are minor ones, all of them metabolically active molecules. Elimination occurs mainly via renal excretion in urine (^∼^85%) [10].

### Pathophysiology And Clinical Presentation

As in many other substance intoxication cases, examinations start with a thorough investigation on the exposure to the substance. This can be done through questioning about prescription medications, over the counter drugs and illicit drugs. The finding of empty pill bottles by witnesses and family members in cases of suicide attempt has a big impact in determining risk of co-ingestion of other substances.

In excessive amounts patients present with a specific caffeine toxidrome [caffeinism] which involves neurological, cardiovascular, gastrointestinal, and renal. These primary toxic effects of caffeine are mediated through multiple mechanisms. Caffeine causes pleiotropic effects depending on the receptors and organs affected, producing paradoxical physiological effects. Antagonism of adenosine receptors by caffeine in the brain are linked with development of altered mentation and seizures. Hypertension is mediated by vasoconstriction though inhibition of the vasodilatory effect of the A2 receptors [11]. Although at physiological doses its inhibition of phosphodiesterase is not significant, toxicity or overdose scenarios make this relevant causing severe hypotension [12]. Caffeine also increases catecholamine levels due to antagonism of pre-synaptic A1 receptors in the sympathetic nerves and the adrenal medulla [13,14]. This adrenergic surge will have clinical consequences such as tachyarrhythmias (b1-stimulation), hypotension, hypokalaemia, leucocytosis, and hyperglycaemia from glycogenolysis (b2-stimulation) and lipolysis (b-3 stimulation). In the renal system caffeine antagonises the A1 receptors in the proximal tubules and prevents sodium reabsorption, finally resulting in increased natriuresis (15).

**Fig. 3:**
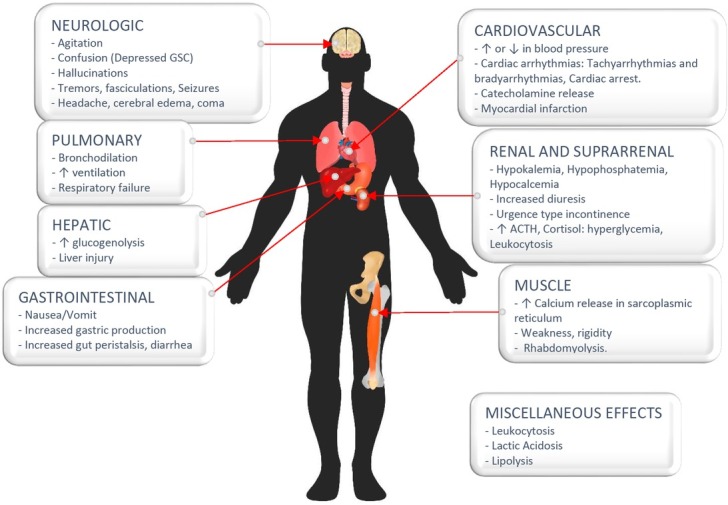
Physiological effects of caffeine intoxication. Adapted from Colin-Benoit et al. [39]

Lactic acidosis is caused by shock induced by hypotension and tissue hypoxia. Rhabdomyolysis has been associated with the calcium release from the sarcoplasmic reticulum of skeletal and cardiac muscle [16, 17]. The full spectrum of caffeine toxicity is summarized in Figure 3.

The average cup of coffee has an estimated of 100mg of caffeine. A greater number of dietary and over the counter supplements and prescription medications also contain caffeine at different doses. Caffeine is rapidly absorbed after oral consumption with clinical effects noticed within 15 minutes and a peak plasma concentration reached after 1 hour of ingestion [18].

Caffeine generally has dose-dependent effects (table 2) and is considered safe at doses of less than 400mg/ day in healthy adults [19]. Toxicity is seen above 400mg/day with sub-lethal dose estimated to be about 7–10 mg/kg of weight in normal adults [20].

**Table 2:** Toxic and lethal caffeine doses and concentrations.

Dose effects of caffeine	Dose
Toxic dose	2 g
Toxic concentration	15 mg/L
Lethal dose	10-20 g
Lethal concentration	80 mg/L

There is no agreement upon the lethal dose of caffeine, however serum concentrations above 80 mg/L are considered lethal, these levels are typically seen after ingestions exceeding 5g. A recent review showed a mean caffeine concentration of 187 ± 96 mg/L with ranges between 33–567 mg/L in patients who died from caffeine intoxication [21]. Nevertheless, there might be individuals who can have serious toxicity and lethality below this range [22,23].

Cardiovascular events are the most common cause of death in patients with caffeine intoxication. The most common mechanisms are rhythm disturbances, with ventricular fibrillation the most frequently described. Myocardial infarction caused by coronary artery vasospasm has also been proposed as one of the causes [21,24].

We did an extensive literature search and review and to date there were 21 well-documented reports of caffeine overdose in adults treated with dialysis techniques. Details pertaining to the serum caffeine levels, clinical presentation and treatment modalities were reviewed and extracted focusing on cardiovascular complications (Table 3). Studies in paediatric populations, non-human research, reviews, laboratory investigations and post-mortem analysis were excluded.

**Table 3:** Cases of caffeine intoxication treated with hemodialysis.

Age/ Gender	Arrhythmias	Laboratory abnormalities	Treatment	Caffeine level (mg/L)	Outcome
37/F	SVT, PVCs, VT, VF	Leukocytosis, hyperglycemia, meta- bolic acidosis	Hemoperfusion, gastric lavage, activated charcoal, norepinephrine, dopamine	199	Recovery [25]
21/F	PAC, PVCs, VT, VF	Metabolic acidosis, Hyponatremia, hypokalemia, hyperglycemia,	Hemoperfusion, activated charcoal	297	Recovery [26]
21/M	None reported	Rhabdomyolysis, renal failure, Hyper- glycemia, hypokalemia	Peritoneal dialysis	Not reported	Recovery [27]
36/-		Rhabdomyolysis, renal failure,	Hemodialysis	-	Recovery [28]
41/M	Wide complex tachy- cardia, bradycardia, 3rd degree AV block, asystole, PEA.	Leukocytosis, hypokalemia, hypergly- cemia, Metabolic acidosis, Rhabdo- myolysis	Hemodialysis and norepineph- rine, dopamine, epinephrine, Vasopressin	405	Recovery [29]
18/M	EKG: Prolonged QTc, ST abnormalities, SVT, PEA,	Leukocytosis, hypokalemia,	Hemodialysis, Phenylephrine and lidocaine infusions.	0.725	Recovery [30]
39/F	Broad complex tachycardia	Lactic acidosis, metabolic acidosis, hypokalemia, hyperglycemia, Rhabdo- myolysis	Activated charcoal, Epineph- rine, dobutamine, CVVHF, hemodialysis	574	Recovery [31]
43/M	None reported	Leukocytosis, hypokalemia, hyper- glycemia, Lactic acidosis, AKI, renal failure	Hemodialysis, Activated char- coal, gastric lavage, Pressors.	60	Recovery [32]
36/M	None reported	No mention	Hemodialysis, Activated char- coal, gastric lavage.	66	Recovery [32]
27/F	VF, Cardiac arrest	Hypokalemia, metabolic acidosis,	Hemofiltration	Not reported	Recovery [33]
42/M	None reported	Leukocytosis, Rhabdomyolysis, Hyper- kalemia, renal failure	Hemodialysis	Not reported	Recovery [34]
44/M	VF, Cardiac arrest	Leukocytosis, hyperglycemia, Rhabdo- myolysis, Troponemia,	ECMO, CVVHF	145.2	Death [35]
32/M	VT	Hypokalemia	Hemoperfusion and hemodi- alysis	200-250	Recovery[36]
50/F	EKG: VT, torsades des pointes, VF, ar- rest, prolonged QTc	Metabolic acidosis, Lactic acidosis, hypokalemia	Norepinephrine and epineph- rine, amiodarone, gastric lavage and charcoal, infusion of intralipid, and CVVHF	311	Recovery [37]
36/M	Sinus tachycardia, PACs,	Leukocytosis, hypokalemia, hypergly- cemia, AKI, rhabdomyolysis.	Hemodialysis	80.16	Recovery [38]
21/M	EKG: Sinus tachycar- dia, RBBB	Leukocytosis, hypokalemia, hyper- glycemia, Elevated CKP, Metabolic acidosis, lactic acidosis	Hemodialysis and hemofiltra- tion, Amiodarone.	150	Recovery [39]
24/M	Sinus tachycardia	Rhabdomyolysis, renal failure, leuko- cytosis, hypokalemia, lactic acidosis.	Hemodialysis, activated char- coal, vasopressors	70	Recovery [40]
43/F	EKG: Wide QRS tachycardia, torsades des pointes, VF. Car- diac arrest, AFib	Lactic acidosis, Metabolic acidosis, hypokalemia, AKI, Rhabdomyolysis, hypoglycemia	ECMO, Continuous hemody- alisis, activated charcoal, lipid emulsion, Vasopressors.	251.9	Recovery [41]
69/M	EKG: SVT, PVC, Bigeminy	Hypokalemia, Rhabdomyolysis, Meta- bolic acidosis, Lactic acidosis	Hemodialysis, Esmolol,	254	Recovery [42]
26/F	EKG: polymorphic VT	metabolic acidosis, Lactic acidosis, hypokalemia	Hemodialysis, intravenous lipid, vasopressors,	147.1	Recovery [43]
43/M	EKG: Wide QRS com- plex tachycardia	Hypokalemia, lactic acidosis	ECMO, Hemodialysis	66	Recovery [44]
-/-	EKG: SVT	Hypokalemia, Metabolic acidosis, lactic acidosis, elevated CPK,	Hemodyalisis, activated char- coal.	118.06	Recovery [45]

Afib: Atrial fibrillation; AKI; Acute kidney injury; CVVHF: Continuous veno-venous hemodiafiltration; ECMO: Extracorporeal membrane oxygenation; PAC: Premature atrial contraction; PEA: Pulseless electrical activity; PVC: Premature ventricular contraction; SVT: Supraventricular tachycardia; VF: Ventricular fibrillation; VT: Ventricular tachycardia

On this review we also mentioned the wide set of treatment strategies have been proposed for the acute management of caffeine intoxication. A common step is the supportive treatment with intravenous fluid resuscitation and the use of potassium chloride and sodium bicarbonate. Symptomatic treatment with benzodiazepines and propofol is a reasonable option in the acute setting where agitation and seizures arise [46].

Decontamination using activated charcoal is considered essential by some authors and should be used in patients presenting within a reasonable time frame of around 1-2 hours [17, 31, 32, 37, 41, 45].

The use of intralipid infusion has been shown to be effective used either alone or in combination with haemodialysis.

Intravenous lipid emulsion is proposed to act by removing of caffeine molecules (an amphiphilic substance) from target organs such as the heart and brain; the so-called “lipid sink.” acting by removal of caffeine molecules (amphiphilic substance) from target organs such as the heart and brain [17, 37, 41, 43].

The first case of caffeine toxicity we found in our review of the literature treated with hemoperfusion was by Zimmerman et al, in the 1980s [25]. Since then, many others have described the use of several dialysis techniques in the management of acute caffeine intoxication [26, 27, 28, 29, 30, 31, 32, 33, 34, 35, 36, 37, 38, 39, 40, 41, 42, 43, 44, 45]. In the reported cases the most common acid base disorder was metabolic acidosis with elevated lactic acid. Other laboratory findings included leucocytosis, hyperglycaemia, hypokalaemia, rhabdomyolysis, acute kidney injury (AKI) and elevated troponins.

Multiple arrhythmias were reported including sinus tachycardia, supraventricular tachycardia [25, 30, 39, 42, 44], bradycardia and 3^rd^ degree AV block [25], PVCs and bigeminy [42], ventricular tachycardia [31, 36, 37, 43, 44], Torsades des pointes [37, 41], and ventricular fibrillation [25, 26, 33, 35, 37, 41].

In the critical and acute phases of intoxication a rapid and effective therapy with intermittent haemodialysis seems effective, with dramatic changes in both clinical status of the patients and outcomes.

Due caffeine’s low molecular weight (194 g/mol), relatively small volume of distribution (0.6–0.8 L/ kg) and the low fraction bound to plasma proteins (10% to 35%) [17], it is a good candidate for extrarenal purification by conventional haemodialysis techniques. Both hemoperfusion and haemodialysis have proven to be successful, with short courses of intermittent HD almost completely reversing the cardiac and neurological signs of caffeine intoxication. Hemoperfusion has been used in the past but its use is limited by practical limitations. Use of other modalities such as continuous veno-venous hemodiafiltration (CVVHF) are adequate options for the removal of excess fluid or uremic toxins. Studies shows mixed results for the use of CVVHF for the treatment of caffeine intoxication, although it seems less effective than HD, there was insufficient data to estimate the clearance values to compare each modality [31, 35, 37].

Extra Corporeal Membrane Oxygenation (ECMO) is one of the newest tools in management of patients with severe cardiovascular and pulmonary dysfunction. The use of veno-arterial ECMO (i.e., VA-ECMO) in patients with severe drug overdose and profound cardiac toxicity has been also reported with positive results. It allows an increase in mean arterial pressure to permit the concomitant use of haemodialysis in patients with caffeine toxicity [35, 41, 44].

In the cases reviewed, most of the articles consisted of a single case of caffeine-related overdose, whereas other included 2-3 patients [32, 38]. One article described the use of haemodialysis in only one of the three cases reported [38]. The time between caffeine ingestion and the establish dialysis was reported in most of the cases. Lack of serial monitoring of caffeine levels was a common denominator in the cases we reviewed.

The current case herein presented had one of the highest reported degrees of caffeine overdose, with approximately 50000 mg of caffeine ingested (387mg/ kg). Our patient presented with progressive cardiovascular and neurological decline which could have progressed given this large dose of caffeine. A multidisciplinary team including toxicology, nephrology, and critical care physicians stabilized the patient and prevented further complications by initiating haemodialysis in a timely manner. The relatively young age of our patient and overall good health could have played one role on the positive outcome.

A wide differential, including caffeine intoxication, and a thorough toxicology work-up is mandatory for patients who present with symptoms of CNS stimulation and vomiting with or without hypokalaemia. Early identification of the typical symptoms may be of critical importance in the emergency room to prompt timely interventions that can be lifesaving in these individuals.

### Limitations

The lack of clinical trials on patients presenting with caffeine intoxication authors cannot recommend which of the treatment options has better outcomes. No clear strategies exist based on the dose of caffeine ingested or time of ingestion.

## Conclusion

Caffeine is a fascinating substance with a wide variety of effects that vary depending on the dose ingested. To this point there is limited understanding of the toxic effects of this drug, and more data is required to determine safe doses especially in sensitive populations. The diagnosis is challenging and mainly depends on the reported ingestion of the substance. Toxidrome symptoms are non-specific and treatment with supportive therapy and decontamination is key. Toxin removal through haemodialysis has shown to be clearly effective with benefits seeing shortly after its initiation.
